# P49 Determinants of trends in reported antibiotic use for sick children from LMICs (2005–17): analysis of 132 national surveys from 73 countries

**DOI:** 10.1093/jacamr/dlac004.048

**Published:** 2022-02-16

**Authors:** Gbemisola Allwell-Brown, Laith Hussain-Alkhateeb, Maquins Odhiambo Sewe, Freddy Kitutu, Susanne Strömdahl, Andreas Mårtensson, Emily White Johansson

**Affiliations:** Uppsala University, Sweden; Gothenburg University, Sweden; Umeå University, Sweden; Makerere University, Uganda; Uppsala University, Sweden; Uppsala University, Sweden; Uppsala University, Sweden

## Abstract

**Objectives:**

To analyse trends in reported antibiotic use for children aged <5 years with fever, diarrhoea or cough with fast or difficult breathing (outcome) from low- and middle-income countries (LMICs) during 2005–17, disaggregated by the following user characteristics: rural/urban residence, maternal education, household wealth and healthcare source visited.

**Methods:**

Based on 132 Demographic and Health Surveys and Multiple Indicator Cluster Surveys from 73 LMICs, the outcome, disaggregated by user characteristics for all country-years was estimated using a hierarchical Bayesian linear regression model.

**Results:**

Across LMICs during 2005–17, the greatest relative increases in the outcome occurred in rural areas, poorest quintiles and least educated populations, particularly in low-income countries and South-East Asia. In low-income countries, rural areas had a 72% relative increase from 17.8% [uncertainty interval (UI): 5.2%–44.9%] in 2005 to 30.6% (11.7%–62.1%) in 2017, compared with a 29% relative increase in urban areas from 27.1% (8.7%–58.2%) in 2005 to 34.9% (13.3%–67.3%) in 2017. Despite these increases, the outcome was consistently highest in urban areas, wealthiest quintiles, and populations with the highest maternal education.

**Conclusions:**

These estimates suggest that the increasing reported antibiotic use for sick children aged <5 years in LMICs during 2005–17 was driven by gains among groups often underserved by formal health services.

